# Supplementary material for: A deeper understanding of system interactions can explain contradictory field results on pesticide impact on honey bees
Source: Nat Commun. 2022 Sep 29;13:5720. doi: 10.1038/s41467-022-33405-7 (PMC9523045; doi:10.1038/s41467-022-33405-7)
Supplement: Supplementary file 1 — Supplementary Information [file 41467_2022_33405_MOESM1_ESM.pdf]

Supplementary Information for

A deeper understanding of system interactions can explain contradictory field results on pesticide impact on honey bees.

Dimitri Breda, Davide Frizzera, Giulia Giordano, Elisa Seffin, Virginia Zanni, Desiderato Annoscia, Christopher J. Topping, Franco Blanchini*, Francesco Nazzi*

*Corresponding author. Email: francesco.nazzi@uniud.it, franco.blanchini@uniud.it

Supplementary Results

According to the proposed conceptual model describing the influence of several stress factors on bee health, we find through the analysis of the equilibria (see Supplementary Methods) that whenever the absolute deviation from desired temperature is small enough:

- the subsystem of honey bees and toxic compounds is monostable (Proposition 2);
- the subsystem of honey bees and parasites is monostable (Proposition 3);
- the subsystem of honey bees, toxic compounds and parasites is monostable (Proposition 4);
- the subsystem of honey bees and viruses can be multistable, a circumstance occurring only if the concerned viruses are immuno-suppressive (Theorem 5).

In particular, according to Theorem 5, bistability in terms of high and low bee health can arise in a given interval of immune suppression capacity, below which the system is monostable at high bee health and above which the system is monostable at low bee health. In the range of bistability, the final state depends on the system initial conditions and minimal differences in these conditions can decide whether the final state is at high or low bee health.

Supplementary Methods

First to proceed, we note that Fig. 2 and Supplementary Fig. 2 and 3 were produced with custom codes developed with the software Mathematica (version 11.3.0.0 run on Mac OS X 10.11.6 MacBook Pro late 2013); Fig. 4 was produced with custom codes developed with the software MATLAB (version R2019a run on Mac OS X 11.6.1 MacBook Pro 2020). All the codes are freely available[26], also at `http://cdlab.uniud.it/software` under the heading `BeeStability`. Moreover, for the reader's convenience, we recall the model from the main text:

$$\begin{cases} \tau_{HB}\dot{x}_{HB} = -\delta_{HB}x_{HB} + g_{TC}(x_{TC}) + g_{VA}(x_{VA}) + g_{VI}(x_{VI}) \\ \qquad\qquad + \overline{f}_{S,C}(u_S, u_C, x_{TC}, x_{VA}) + \overline{f}_P(u_P, x_{TC}) + \underline{f}_{HB}(u_T) \\ \tau_{TC}\dot{x}_{TC} = -\delta_{TC}x_{TC} + g_{HB}(x_{HB}) \\ \tau_{VA}\dot{x}_{VA} = -\delta_{VA}x_{VA} + h_{VA}(x_{HB}, x_{TC}, \varepsilon x_{VI}) + \underline{f}_{VA}(u_T) \\ \tau_{VI}\dot{x}_{VI} = -\delta_{VI}x_{VI} + h_{VI}(x_{HB}, x_{TC}, \varepsilon x_{VI}). \end{cases} \tag{1}$$

As it can be deduced from the proof of Proposition 1 in the section "Structural analysis of the bee health model" of the main text, system (1) is monotone (since the matrix M is Metzler[1]). Monotone systems can exhibit multiple equilibria and hence multistability[1]. To investigate whether this is the case for system (1) we must specify the form of the functions g, $\overline{f}$, $\underline{f}$ and h. The following choices are among the simplest capable of portraying the desired features (monotonicity, convexity, saturation, etc.). As such, they reasonably represent the concerned

interactions without compromising the generality of the conceptual model. We thus focus on

$$
\begin{cases}
\tau_{HB}\dot{x}_{HB} = -x_{HB} + \dfrac{\alpha}{1+\beta x_{TC}} + \dfrac{\mu}{1+\nu x_{VA}} + \dfrac{\sigma}{1+\rho x_{VI}} + \dfrac{c_0 u_S(1+c_1 u_C)}{1+c_2 x_{VA}+c_3 x_{TC}} \\
\qquad\qquad + \dfrac{c_4 u_P}{1+c_5 x_{TC}} - c_6 u_T, \\
\tau_{TC}\dot{x}_{TC} = -x_{TC} + \dfrac{\xi}{1+\omega x_{HB}}, \\
\tau_{VA}\dot{x}_{VA} = -x_{VA} + \dfrac{\eta}{1+\dfrac{\lambda x_{HB}}{1+\psi x_{TC}+\vartheta\varepsilon x_{VI}}} - c_7 u_T, \\
\tau_{VI}\dot{x}_{VI} = -x_{VI} + \dfrac{\gamma}{1+\dfrac{\varphi x_{HB}}{1+\chi x_{TC}+\varepsilon x_{VI}}},
\end{cases}
\tag{2}
$$

where we set $\delta = 1$ for each state variable without loss of generality.

The search for equilibria passes through annihilating the right-hand side, leading to the algebraic system

$$
\begin{cases}
x_{HB} = \dfrac{\alpha}{1+\beta x_{TC}} + \dfrac{\mu}{1+\nu x_{VA}} + \dfrac{\sigma}{1+\rho x_{VI}} + \dfrac{c_0 u_S(1+c_1 u_C)}{1+c_2 x_{VA}+c_3 x_{TC}} + \dfrac{c_4 u_P}{1+c_5 x_{TC}} - c_6 u_T, \\
x_{TC} = \dfrac{\xi}{1+\omega x_{HB}}, \\
x_{VA} = \dfrac{\eta}{1+\dfrac{\lambda x_{HB}}{1+\psi x_{TC}+\vartheta\varepsilon x_{VI}}} - c_7 u_T, \\
x_{VI} = \dfrac{\gamma}{1+\dfrac{\varphi x_{HB}}{1+\chi x_{TC}+\varepsilon x_{VI}}}.
\end{cases}
\tag{3}
$$

A full analysis of (3) is not immediate. We thus proceed by analyzing different subsystems, finally showing that the presence of pathogens impairing the immune system is a necessary condition for multistability. In the sequel, with the term *equilibrium* we implicitly refer to *positive* values of the state variables considered in each specific subsystem, the only biologically meaningful. In this sense, all the following results share the common assumption of having u_T sufficiently small. This assumption prevents the honey bee health at equilibrium from being

null or negative (and the same for the parasites when present). We stress that the assumption is necessary because a large u_T would be disruptive and it would prevent any survival possibility.

We start by considering the case in which the only stress factor is a toxic compound.

Proposition 2*:* If u_T is sufficiently small, the subsystem of honey bees and toxic compounds (i.e., first and second equation in (2) with $\nu = \rho = c_2 = 0$) has a unique equilibrium, globally asymptotically stable.

Proof: Reduce system (2) to

$$\begin{cases} \tau_{HB}\dot{x}_{HB} = -x_{HB} + \dfrac{\alpha}{1+\beta x_{TC}} + \mu + \sigma + \dfrac{c_0 u_S(1+c_1 u_C)}{1+c_3 x_{TC}} + \dfrac{c_4 u_P}{1+c_5 x_{TC}} - c_6 u_T, \\ \tau_{TC}\dot{x}_{TC} = -x_{TC} + \dfrac{\xi}{1+\omega x_{HB}}. \end{cases} \tag{4}$$

It is immediate to recover the nullcline[†] for x_{HB}

$$x_{HB} = \frac{\alpha}{1+\beta x_{TC}} + \mu + \sigma + \frac{c_0 u_S(1+c_1 u_C)}{1+c_3 x_{TC}} + \frac{c_4 u_P}{1+c_5 x_{TC}} - c_6 u_T \tag{5}$$

and the nullcline for x_{TC}

$$x_{HB} = \frac{1}{\omega}\left(\frac{\xi}{x_{TC}} - 1\right). \tag{6}$$

Equating the two leads to finding that possible equilibria are characterized by $x_{TC} = y$ for y any positive solution of

$$LHS(y) = RHS(y), \tag{7}$$

where

$$LHS(y) := \left[\xi - \omega y\left(\frac{1}{\omega} + \mu + \sigma - c_6 u_T\right)\right](1+\beta y)(1+c_3 y)(1+c_5 y)$$

[†]A nullcline for a state variable of a planar system is a curve in the phase plane along which that variable is locally constant. As such it is obtained by annihilating the relevant derivative and hence the right-hand side of the corresponding ODE. It follows that equilibria are intersection points of nullclines of different state variables.

and

$$RHS(y) := \omega y \big[\alpha(1+c_3y)(1+c_5y) + c_0u_S(1+c_1u_C)(1+\beta y)(1+c_5y) \\ + c_4u_P(1+\beta y)(1+c_3y)\big].$$

On the one hand, LHS is a fourth degree polynomial in y, with negative leading term, three negative roots and one positive root

$$y_+ := \frac{\xi}{1+\omega(\mu+\sigma-c_6u_T)}.$$

The fact that y_+ is positive follows by assuming $u_T < (\mu+\sigma)/c_6$, condition guaranteeing that the equilibrium coordinate x_{HB} is positive (indeed, the minimum such value is $\mu+\sigma-c_6u_T$ when $x_{TC} \to +\infty$ as it follows from (5)). On the other hand, RHS is a third degree polynomial in y, with positive leading term and a root in 0. By factoring this trivial root out, the remaining second degree polynomial has positive coefficients, hence no positive roots (as it follows by applying Descartes' rule of signs[‡]). It follows that there exists a unique positive solution y of (7), being indeed $LHS(0) = \xi > 0 = RHS(0)$.

Global stability of the corresponding equilibrium immediately follows by analyzing the nullclines. In the phase plane (x_{TC}, x_{HB}), orbits through points above (5) move downward (since $\dot{x}_{HB} < 0$), while orbits through points below move upward (since $\dot{x}_{HB} > 0$). On the other hand, orbits through points to the right of (6) move leftward (since $\dot{x}_{TC} < 0$), while orbits through points to the left move rightward (since $\dot{x}_{TC} > 0$). Their unique intersection point (i.e., the equilibrium) is thus globally attracting, Supplementary Fig. 2a. This ends the proof of Proposition 2.

Going back to the general model (1), from the proof above we can conclude that any reasonable choice of the functions g, $\overline{f}$ and $\underline{f}$ satisfying the required assumptions of smoothness, monotonicity and convexity can determine uniqueness and global stability.

[‡]The number of positive roots of a polynomial is at most the number of sign changes in the series of ordered coefficients in its canonical expression.

The second result concerns the case in which the only stress factor is due to the presence of parasites.

Proposition 3*:* If u_T is sufficiently small, the subsystem of honey bees and parasites (i.e., first and third equation in (2) with $\beta = \rho = \psi = \theta = c_3 = c_5 = 0$) has a unique equilibrium, globally asymptotically stable.

Proof: Similarly to the proof of Proposition 2 we reduce (2) to

$$\begin{cases} \tau_{HB}\dot{x}_{HB} = -x_{HB} + \alpha + \dfrac{\mu}{1+\nu x_{VA}} + \sigma + \dfrac{c_0 u_S(1+c_1 u_C)}{1+c_2 x_{VA}} + c_4 u_P - c_6 u_T, \\ \tau_{VA}\dot{x}_{VA} = -x_{VA} + \dfrac{\eta}{1+\lambda x_{HB}} - c_7 u_T, \end{cases} \tag{8}$$

and recover the nullcline for x_{HB}

$$x_{HB} = \alpha + \frac{\mu}{1+\nu x_{VA}} + \sigma + \frac{c_0 u_S(1+c_1 u_C)}{1+c_2 x_{VA}} + c_4 u_P - c_6 u_T \tag{9}$$

and the nullcline for x_{VA}

$$x_{HB} = \frac{1}{\lambda}\left(\frac{\eta}{x_{VA}+c_7 u_T} - 1\right). \tag{10}$$

Equating the two leads to finding that possible equilibria are characterized by $x_{VA} = z$ for z any positive solution of

$$LHS(z) = RHS(z), \tag{11}$$

where

$$LHS(z) := \{\eta - (z + c_7 u_T)[1 + \lambda(\alpha + \sigma + c_4 u_P - c_6 u_T)]\}\,(1+\nu z)(1+c_2 z)$$

and

$$RHS(z) := \lambda(z + c_7 u_T)[\mu(1+c_2 z) + u_S(1+c_1 u_C)(1+\nu z)].$$

On the one hand, LHS is a third degree polynomial in z, with negative leading term, two negative roots and one positive root

$$z_+ := \frac{\eta - c_7 u_T[1+\lambda(\alpha+\sigma+c_4 u_P - c_6 u_T)]}{1+\lambda(\alpha+\sigma+c_4 u_P - c_6 u_T)}.$$

In fact, from (9) we have $x_{HB} \in (x_{HB,\min}, x_{HB,\max})$ for

$$x_{HB,\min} := \alpha + \sigma + c_4 u_P - c_6 u_T,$$

obtained by letting $x_{VA} \to +\infty$, and

$$x_{HB,\max} := \alpha + \mu + \sigma + c_0 u_S(1 + c_1 u_C) + c_4 u_P - c_6 u_T$$

obtained by choosing $x_{VA} = 0$. Then, by assuming u_T sufficiently small (in particular, smaller than $(\alpha + \sigma + c_4 u_P)/c_6$ so as to guarantee $x_{HB,\min} > 0$), we get

$$\eta - c_7 u_T[1 + \lambda(\alpha + \sigma + c_4 u_P - c_6 u_T)] = \eta - c_7 u_T[1 + \lambda x_{HB,\min}] > \eta - c_7 u_T[1 + \lambda x_{HB}] > 0$$

with the last inequality holding true, otherwise x_{VA} would not be positive from (10). On the other hand, RHS is a second degree polynomial in z, with positive leading term and two negative roots. Since

$$\begin{aligned} c_7 u_T < \frac{\eta}{1 + \lambda x_{HB,\max}} &\Leftrightarrow c_7 u_T < \frac{\eta}{1 + \lambda(\alpha + \mu + \sigma + c_0 u_S(1 + c_1 u_C) + c_4 u_P - c_6 u_T)} \\ &\Leftrightarrow c_7 u_T[1 + \lambda(\alpha + \mu + \sigma + c_0 u_S(1 + c_1 u_C) + c_4 u_P - c_6 u_T)] < \eta \\ &\Leftrightarrow \lambda c_7 u_T[\mu + c_0 u_S(1 + c_1 u_C)] < \eta - c_7 u_T[1 + \lambda(\alpha + \sigma + c_4 u_P - c_6 u_T)] \\ &\Leftrightarrow RHS(0) < LHS(0) \end{aligned}$$

holds true for u_T sufficiently small, it follows that there exists a unique positive solution z of (11).

Finally, that the corresponding equilibrium is globally asymptotically stable follows similarly as in the proof of Proposition 2, Supplementary Fig. 2b. This ends the proof of Proposition 3.

A comment similar to the one placed soon below the proof of Proposition 2 holds here as well: any reasonable choice of the functions g, h, $\overline{f}$ and $\underline{f}$ in the first and third equation of the general model (1) satisfying the required assumptions can ensure uniqueness and global stability.

The third result concerns the case in which the stress factor is due to the presence of both toxic compounds and parasites. As the relevant subsystem is not planar anymore, the proof requires slightly different and more geometrical considerations.

Proposition 4*:* If u_T is sufficiently small, the subsystem of honey bees, toxic compounds and parasites (i.e., first, second and third equation in (2) with $\rho = \theta = 0$) has a unique equilibrium, globally asymptotically stable.

Proof: Reduce (3) to

$$\begin{cases} x_{HB} = \dfrac{\alpha}{1+\beta x_{TC}} + \dfrac{\mu}{1+\nu x_{VA}} + \sigma + \dfrac{c_0 u_S(1+c_1 u_C)}{1+c_2 x_{VA}+c_3 x_{TC}} + \dfrac{c_4 u_P}{1+c_5 x_{TC}} - c_6 u_T, \\ x_{TC} = \dfrac{\xi}{1+\omega x_{HB}}, \\ x_{VA} = \dfrac{\eta}{1+\dfrac{\lambda x_{HB}}{1+\psi x_{TC}}} - c_7 u_T \end{cases} \tag{12}$$

and note that u_T must be sufficiently small similarly to the proofs of Proposition 2 and Proposition 3. Recover x_{HB} from the last equation as

$$x_{HB} = \frac{1+\psi x_{TC}}{\lambda}\left(\frac{\eta}{x_{VA}+c_7 u_T} - 1\right). \tag{13}$$

In order to prove uniqueness, in the phase space (x_{TC}, x_{VA}, x_{HB}) we first consider from the first of (12) the family of curves $x_{HB} = f_1(x_{TC}; x_{VA})$ describing x_{HB} as a function of x_{TC} parametrized along x_{VA} (i.e., imagine "slices" of the surface f_1 w.r.t. the variable x_{VA}): each curve is smooth, bounded, positive and convex, monotonically decreasing from

$$f_1(0; x_{VA}) = \alpha + \frac{\mu}{1+\nu x_{VA}} + \sigma + \frac{c_0 u_S(1+c_1 u_C)}{1+c_2 x_{VA}} + c_4 u_P - c_6 u_T$$

asymptotically to

$$f_1(+\infty; x_{VA}) = \frac{\mu}{1+\nu x_{VA}} + \sigma - c_6 u_T$$

and both these quantities are monotonically decreasing w.r.t. x_{VA}, Supplementary Fig. 3a. We consider as well the family of curves $x_{HB} = f_2(x_{TC}; x_{VA})$ describing x_{HB} as a function of x_{TC} parametrized along x_{VA} through (13): each curve is a line of slope

$$f_2'(x_{HB}; x_{VA}) = \frac{\psi}{\lambda}\left(\frac{\eta}{x_{VA} + c_7 u_T} - 1\right)$$

and is monotonically increasing from

$$f_2(0; x_{VA}) = \frac{1}{\lambda}\left(\frac{\eta}{x_{VA} + c_7 u_T} - 1\right)$$

and both these quantities are again monotonically decreasing w.r.t. x_{VA}, Supplementary Fig. 3b. It follows that for any fixed positive value of x_{VA}, there is a unique intersection point between f_1 and f_2 whenever $f_1(0; x_{VA}) > f_2(0; x_{VA})$.

Now, we know from Proposition 3 that in the plane (x_{VA}, x_{HB}) (i.e., $x_{TC} = 0$) the curves $g_1(x_{VA}) := f_1(0; x_{VA})$ and $g_2(x_{VA}) := f_2(0; x_{VA})$ have a unique intersection with positive coordinate x_{VA}. Beyond this intersection g_1 is above g_2 and the former decreases more slowly than the latter, so that the above mentioned unique intersection point between f_1 and f_2 decreases with x_{VA}, Supplementary Fig. 3c.

Finally, by following arguments similar to those used in the proof of Proposition 2 it is not difficult to show that, when projected into the plane (x_{TC}, x_{HB}), the locus of such points forms a decreasing curve which intersects the curve given by the second of (12) only once, thus proving uniqueness, Supplementary Fig. 3d.

Global asymptotic stability follows by considerations similar to those argued in the proof of both Proposition 2 and Proposition 3, with the only difference that now the nullclines are surfaces rather than curves. This ends the proof of Proposition 4.

Again, concerning the general model (1), any reasonable choice of the functions g, h, $\overline{f}$ and $\underline{f}$ satisfying the required assumptions can ensure uniqueness and global stability.

Now we state and prove the main result, concerning the role of the immune suppression capacity of the pathogen when interacting with the bee in the absence of other stressors. First let us define

$$a(\varepsilon) := \rho(1 + k\varphi) - \varepsilon(\rho\gamma - 1), \quad b(\varepsilon) := 1 + \varphi(k + \sigma) - \rho\gamma - \varepsilon\gamma, \tag{14}$$

where

$$k := \alpha + \mu + c_0 u_S(1 + c_1 u_C) + c_4 u_P - c_6 u_T$$

is introduced for brevity.

Theorem 5*:* Let u_T be sufficiently small and $a(\varepsilon)$ and $b(\varepsilon)$ be given by (14). If

$$a(\varepsilon) < 0, \quad 0 < b(\varepsilon) < \frac{a^2(\varepsilon)}{3\rho\varepsilon}, \tag{15}$$

then, by varying $\varepsilon \geq 0$, the subsystem of honey bees and pathogens (i.e., first and fourth equation in (2) with $\beta = \nu = \chi = c_2 = c_3 = c_5 = 0$) exhibits two fold bifurcations[§], viz. at $\varepsilon = \varepsilon_{\min}$ and $\varepsilon = \varepsilon_{\max}$ with $0 < \varepsilon_{\min} < \varepsilon_{\max}$, and

(A1) a unique equilibrium, globally asymptotically stable with high bee health, if and only if $\varepsilon < \varepsilon_{\min}$;

(A2) three equilibria, two locally asymptotically stable (with high and low bee health, respectively) and one unstable (with intermediate bee health), if and only if $\varepsilon_{\min} < \varepsilon < \varepsilon_{\max}$;

(A3) a unique equilibrium, globally asymptotically stable with low bee health, if and only if $\varepsilon > \varepsilon_{\max}$.

[§]A fold bifurcation occurs when there exists a value of a system parameter below which, locally, there are no equilibria and above which, always locally, there are two equilibria (which coincide at the bifurcation value), or the other way around[2].

Otherwise, if (15) does not hold, the system always has a unique equilibrium, globally asymptotically stable.

Proof: Again, the necessity of the assumption that u_T is sufficiently small is clear from the proofs of Proposition 2, Proposition 3 and Proposition 4. In the usual manner we recover the nullcline for x_{HB}

$$x_{HB} = k + \frac{\sigma}{1 + \rho x_{VI}} \tag{16}$$

and the nullcline for x_{VI}

$$x_{HB} = \frac{1}{\varphi}\left[\frac{\gamma}{x_{VI}} - 1 + \varepsilon(\gamma - x_{VI})\right]. \tag{17}$$

Equating the two leads to finding that possible equilibria are characterized by $x_{VI} = v$ for v any positive root of the third-degree polynomial

$$p(v;\varepsilon) := \rho\varepsilon v^3 + a(\varepsilon)v^2 + b(\varepsilon)v - \gamma \tag{18}$$

parametrized by ε.

We start from the case $\varepsilon = 0$, by observing that $p(v;0)$ is a parabola heading upward and $p(0;0) = -\gamma < 0$ implies the existence of one positive root. This root is unique since $a(0) > 0$, so that $p(v;0)$ has only one sign change independently of the sign of $b(0)$ and the conclusion follows by applying Descartes' rule of signs.

Assume then $\varepsilon > 0$. We show that $b(\varepsilon) \leq 0$ implies uniqueness (the same reasoning is valid also for $a(\varepsilon) \geq 0$, the proof of which we thus omit). Indeed, in this case $p(v;\varepsilon)$ has only one sign change (independently of the sign of $a(\varepsilon)$), hence at most one positive root again according to Descartes' rule of signs, and it has exactly one positive root since $p(0;\varepsilon) = -\gamma \leq 0$ and the leading term is positive.

So $b(\varepsilon) > 0$ is a necessary condition to violate uniqueness, which we assume to hold true in the following. By considering only simple roots for the time being (i.e., those for which $p'(v;\varepsilon) \neq 0$), uniqueness is lost only in favor of three positive roots. For this to be the case there must be three sign changes in the coefficients of $p(v;\varepsilon)$, which is possible only if $a(\varepsilon) < 0$. Therefore this constraint is a second necessary condition for non-uniqueness. A third necessary condition is given by the existence of a local minimum and a local maximum, which is equivalent to $p'(v;\varepsilon) = 3\rho\varepsilon v^2 + 2a(\varepsilon)v + b(\varepsilon)$ having two distinct zeros. Hence it must be $b(\varepsilon) < a^2(\varepsilon)/(3\rho\varepsilon)$, thus completing (15).

The final result under the last condition follows by observing first that $p(\gamma;\varepsilon)$, $p'(\gamma;\varepsilon)$ and $p''(\gamma;\varepsilon)$ are all positive, so that the rightmost positive root of $p(v;\varepsilon)$ as well as its local minimum and maximum lie all to the left of γ and second that when $\rho\gamma > 1$ (which is necessarily implied by $a(\varepsilon) < 0$) $p(v;\varepsilon)$ decreases with ε when $v < \gamma$, in fact this implies

$$\frac{\partial p(v;\varepsilon)}{\partial \varepsilon} = \rho v^3 + (1-\rho\gamma)v^2 - v\gamma < 0.$$

The outcome is summarized in Fig. 2d, which illustrates the behavior of $p(v;\varepsilon)$ under (15) for increasing ε (i.e., from top to bottom $\varepsilon = 1.55$: one equilibrium at high bee health; $\varepsilon = \varepsilon_{\min} \approx 1.56519$: one equilibrium at high bee health and fold bifurcation at low bee health; $\varepsilon = 1.58$: three equilibria; $\varepsilon = \varepsilon_{\max} \approx 1.61591$: one equilibrium at low bee health and fold bifurcation at high bee health; $\varepsilon = 1.63$: one equilibrium at low bee health; see Supplementary Tab. **4** for the values of the parameters), and fully justifies the thesis with the understanding that a low level of x_{VI} (i.e., a low value of v) corresponds to a high level of bee health. In particular, when $\varepsilon = 0$ the only positive root is to the left of the local maximum (low v, high bee health). When ε increases a first value is encountered, i.e., $\varepsilon_{\min}$, at which the local minimum touches the v-axis from above, generating two new positive roots to the right of the existing one (hence with intermediate and low bee health). By increasing ε further these three positive roots continue to

exist, with the intermediate one moving leftward to the leftmost one, until at a second value for ε, i.e., $\varepsilon_{\max}$, these two roots collide and coincide with the local maximum to then disappear, leaving alone the positive rightmost root (high v, low health). This ends the proof of Theorem 5.

As noted in the proof above, $a(\varepsilon) < 0$ cannot hold true if $\rho\gamma \leq 1$, which is biologically meaningful: when the effect of the pathogen's growth γ combined with the influence ρ on the bee health is sufficiently low, the immune suppression capacity is not effective. Moreover, when $\rho\gamma$ is large enough so that $a(\varepsilon) < 0$ it is not difficult to show that there are choices of the parameter values for which also the second condition in (15) is satisfied, so that multiple equilibria are indeed possible (as in Fig. 2c). In particular, for this to be true also σ must be large enough, which in fact weighs the negative direct effect of pathogens on bee health (through ρ).

To complete the whole analysis, we observe that also the full system (1) with $\varepsilon = 0$ exhibits uniqueness and global stability, as the interaction of pathogens and parasites with the bee are modeled in the same way in this case. Instead, if $\varepsilon > 0$, multistability in the sense of Theorem 5 is possible, as it can be proved by assessing the introduction of toxic compounds and/or parasites on the base of continuity arguments.

Supplementary Tables

Supplementary Table 1: An extract of significant studies on the effect of neonicotinoid insecticides on honey bees carried out under field conditions in the period 2014-2019.

Region	Crop	Insecticide	Bee species	Effect	Reference
Canada	oilseed rape	clothianidin	*Apis mellifera*	No impact	[3]
United Kingdom	oilseed rape	imidacloprid	*Apis mellifera*	Negative effects	[4]
Sweden	oilseed rape	clothianidin	*Apis mellifera*, *Bombus terrestris*	No impact on honey-bees, effects on bumble bees	[5]
France	oilseed rape	thiamethoxam, imidacloprid	*Apis mellifera*	Negative effects at individual level, no impact at colony level	[6]
Germany	oilseed rape	clothianidin	*Apis mellifera*	No impact	[7]
Canada	corn	clothianidin, thiamethoxam, imidacloprid, acetamiprid	*Apis mellifera*	Negative effects	[8]
Hungary, Germany, United Kingdom	oilseed rape	clothianidin, thiamethoxam	*Apis mellifera*, *Bombus terrestris*, *Osmia bicornis*	Honeybees: negative effects (Hungary and United Kingdom) and positive effects (Germany). Bumblebees and Red Mason bee: negative effects	[9]
Sweden	oilseed rape	clothianidin	*Apis mellifera*	No impact	[10]

Supplementary Table 2: **Major interactions in the honey bee system.** Letters refer to arrows in the conceptual model depicted in Fig. 1.

Interaction	from	to	Description	most relevant reference
a	Varroa	Honey bee	The mite subtracts haemolymph ultimately reducing the survival of bees	[11]
b	Virus	Honey bee	Viruses replicate within the bee cells ultimately reducing the survival of bees	[12]
c	Toxic compounds	Honey bee	Toxic compounds can have both lethal and sublethal effects on honey bees	[13]
d	Sub-optimal temperature	Honey bee	Honey bees need a temperature of 34.5 °C for optimal survival; any difference from that value can influence survival, affecting metabolism and other vital functions	[14]
e	Nectar	Honey bee	A sufficient intake of sugars is essential for survival	[15]
f	Pollen	Honey bee	Pollen is an important nutrient and sustains defences against parasites and pathogens	[15]
g	Toxic compounds	Honey bee	Nectar can be contaminated by toxic compounds both from natural and artificial sources which can affect honey bee health	[16]
h	Toxic compounds	Honey bee	Pollen may contain toxic compounds both from natural and artificial sources	[17]
i	Honey bee immune system	Varroa	Clotting and melanization at the feeding site interfere with mite nutrition	[18]
j	Honey bee immune system	Viruses (e.g., DWV)	Antiviral response (e.g., haemocytes, AMPs, RNAi) limits viral replication	[19]
k	Honey bees detoxification system	Toxic compounds	A potent detoxification system may degrade toxic compounds	[20]
l	Low temperature	Intake of sugars	In case of temperature lower than 34.5 °C, bees increase sugar consumption to warm up their body	[21]
m	Virus	Honey bee immune system	Above a certain threshold, DWV can impair NF-kB and thus both antiviral response and melanization	[22]
n	Toxic compounds	Honey bee immune system	Some toxic compounds can impair immunity (e.g., NF-kB)	[23]
o	Varroa	Sugars	Mite infestation reduces sugar consumption	[24]
p	Sub-optimal temperature	Varroa	The mite is adapted to the optimal temperature for honey bees and may suffer from non-optimal temperatures; however, it is extremely rare that brood -where the mite reproduces- can suffer sub-optimal temperatures	[25]

Supplementary Table 3: Properties of the functions of the model of honey bee health, together with a summary of the biological effects they account for and a reference to the conceptual model in Fig. 1.

family of functions	properties	function	model equation in the main text	function properties	biological effect	arrow in Fig. 1
g	smooth, bounded, positive, convex and decreasing to 0	$g_{TC}(x_{TC})$	1	decreasing to 0 w.r.t. x_{TC}	toxic compounds negatively influence honey bee health	c
		$g_{VA}(x_{VA})$	1	decreasing to 0 w.r.t. x_{VA}	parasites negatively influence honey bee health	a
		$g_{VI}(x_{VI})$	1	decreasing to 0 w.r.t. x_{VI}	viruses negatively influence honey bee health	b
		$g_{HB}(x_{HB})$	2	decreasing to 0 w.r.t. x_{HB}	detoxification by the honey bee can reduce the concentration of toxic compounds	k
$\overline{f}$	smooth, bounded, non-negative, concave and increasing w.r.t. u arguments (vanishing only when the first u argument vanishes) while convex and decreasing to 0 w.r.t. x arguments	$\overline{f}_{S,C}(u_S, u_C, x_{TC}, x_{VA})$	1	concave and increasing w.r.t. u_S	sugars from nectar promote honey bee health	e
				concave and increasing w.r.t. u_C	honey bees increase sugar feeding to counteract low temperatures	l
				convex and decreasing w.r.t. x_{TC}	nectar can be contaminated with toxic compounds	g
				convex and decreasing w.r.t. x_{VA}	mite infested honey bees may consume less sugar	o
		$\overline{f}_P(u_P, x_{TC})$	1	concave and increasing w.r.t. u_P	proteins from pollen promote honey bee health	f
				convex and decreasing w.r.t. x_{TC}	pollen can be contaminated with toxic compounds	h
$\underline{f}$	smooth, bounded, non-positive and decreasing (vanishing only when $u = 0$)	$\underline{f}_{HB}(u_T)$	1	decreasing w.r.t. u_T	sub-optimal thermal conditions affect honey bee health	d
		$\underline{f}_{VA}(u_T)$	3	decreasing w.r.t. u_T	lower temperatures can negatively influence parasites	p
h	smooth, bounded, positive, convex and decreasing to 0 w.r.t. the first argument, concave and increasing w.r.t. all the other arguments	$h_{VA}(x_{HB}, x_{TC}, \varepsilon x_{VI})$	3	convex and decreasing to 0 w.r.t. x_{HB}	honey bee immunity negatively influences parasites	i
				concave and increasing w.r.t. x_{TC}	by impairing immunity, toxic compounds can favour parasite feeding	n
				concave and increasing w.r.t. εx_{VI}	by impairing immunity, viruses can favour parasite feeding	m
		$h_{VI}(x_{HB}, x_{TC}, \varepsilon x_{VI})$	4	convex and decreasing to 0 w.r.t. x_{HB}	honey bee immunity negatively influences viruses	j
				concave and increasing w.r.t. x_{TC}	by impairing immunity, toxic compounds can favour viruses	n
				concave and increasing w.r.t. εx_{VI}	by impairing immunity, viruses can favour their own replication	m

Supplementary Table 4: **Values of the parameters in** (2) **for the figures included here and in the main text.** The values of ε used in Fig. 2d are, from top to bottom: 1.55, ≈1.56519, 1.58, ≈1.61591, 1.63.

parameter	Fig. 2a	Fig. 2b	Fig. 2c	Fig. 2d	Supplementary Fig. 2a	Supplementary Fig. 2b	Supplementary Fig. 3
τ_{HB}	1	1	1	1	1	1	1
α	1	1	1	1	1	1	1
β	1	1	1	0	1	0	1
μ	1	1	1	1	1	1	1
ν	1	1	1	0	0	1	1
σ	35	35	35	44	1	1	1
ρ	1	1	1	1.6	0	0	0
c_0	1	1	1	1	1	1	1
u_S	1	1	1	1	1	1	1
c_1	1	1	1	1	1	1	1
u_C	1	1	1	1	1	1	1
c_2	1	1	1	0	0	1	1
c_3	1	1	1	0	1	0	1
c_4	1	1	1	1	1	1	1
u_P	1	1	1	1	1	1	1
c_5	1	1	1	0	1	0	1
c_6	1	1	1	1	1	1	1
u_T	1	1	1	1	1	1	1
τ_{TC}	1	1	1	-	1	-	1
ξ	1	1	1	-	1	-	1
ω	1	1	1	-	1	-	1
τ_{VA}	1	1	1	-	-	1	1
η	20	200	20	-	-	1	-
λ	1	1	1	-	-	1	1
ψ	1	1	1	-	-	0	1
θ	1	1	1	-	-	0	0
ε	0	0	1.34	varying*	-	-	-
c_7	0.1	0.1	0.1	-	-	1	1
τ_{VI}	1	1	1	1	-	-	-
γ	30	200	30	9	-	-	-
φ	1	1	1	1	-	-	-
χ	1	1	1	0	-	-	-

Supplementary Figures

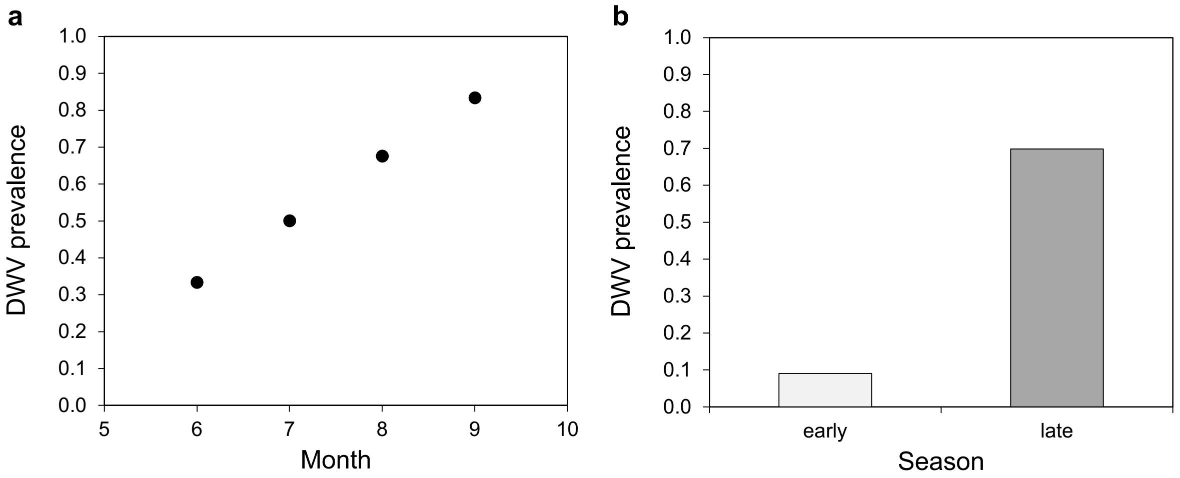

Supplementary Figure 1: **Seasonal variation of DWV infection in control bees.** (**a**) Virus prevalence (proportion of virus infected bees) increases from June to September. (**b**) Before mid July ("early") the majority of bees can be considered as virus free; later ("late") most bees are virus infected.

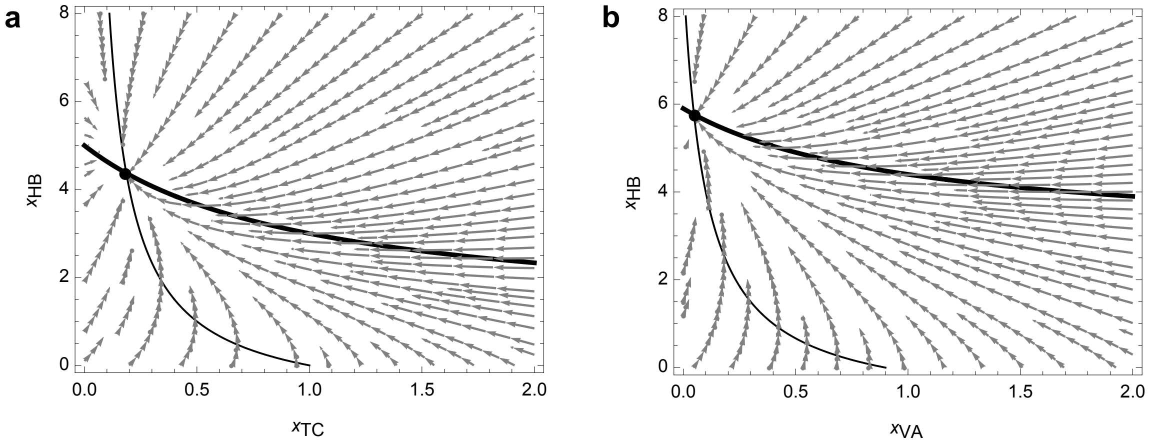

Supplementary Figure 2: **Flow and nullclines.** (**a**) Flow (gray arrows) of system (4) in the phase plane (x_{TC}, x_{HB}), with the nullcline (5) for x_{HB} (thick black), the nullcline (6) for x_{TC} (thin black) and the unique equilibrium (•); (**b**) flow (gray arrows) of system (8) in the phase plane (x_{VA}, x_{HB}), with the nullcline (9) for x_{HB} (thick black), the nullcline (10) for x_{VA} (thin black) and the unique equilibrium (•); see Supplementary Tab. **4** for the values of the parameters.

Supplementary References

1. Hirsch, M. W. & Smith, H. "Chapter 4 Monotone Dynamical Systems" in Handbook of differential equations: ordinary differential equations, Canada, A., Drabek, P. & Fonda, A. Eds. 239-357 (2006).

2. Kuznetsov, Y. A. Elements of applied bifurcation theory, **112** in Applied Mathematical Sciences (Springer-Verlag, 1988).

3. Cutler, G. C., Scott-Dupree, C. D., Sultan, M., McFarlane, A. D., & Brewer, L. A large-scale field study examining effects of exposure to clothianidin seed-treated canola on honey bee colony health, development, and overwintering success. *PeerJ 2*, e652 (2014).

4. Budge, G. E., Garthwaite, D., Crowe, A., Boatman, N. D., Delaplane, K. S., Brown, M. A., Thygesen, H. H. & Pietravalle, S. Evidence for pollinator cost and farming benefits of neonicotinoid seed coatings on oilseed rape. *Sci. Rep.* **5**, 12574 (2015).

5. Rundlöf, M., Andersson, G. K. S., Bommarco, R., Fries, I., Hederström, V., Herbertsson, L., Jonsson, O., Klatt, B. K., Pedersen, T. R., Yourstone, J. & Smith, H. G. Seed coating with a neonicotinoid insecticide negatively affects wild bees. *Nature* **521**, 77-80 (2015).

6. Henry, M., Cerrutti, N., Aupinel, P., Decourtye, A., Gayrard, M., Odoux, J.-F., Pissard, A., Rüger, C. & Bretagnolle, V. Reconciling laboratory and field assessments of neonicotinoid toxicity to honeybees. *Proc. Royal Soc. B* **282**, 1819 (2015).

7. Rolke, D., Fuchs, S., Grünewald, B., Gao, Z. & Blenau, W. Large-scale monitoring of effects of clothianidin-dressed oilseed rape seeds on pollinating insects in Northern Germany: effects on honey bees (Apis mellifera). *Ecotoxicology* **25**, 1648-1665 (2016).

8. Tsvetkov, N., Samson-Robert, O., Sood, K., Patel, H. S., Malena, D. A., Gajiwala, P. H., Maciukiewicz, P., Fournier, V. & Zayed, A. Chronic exposure to neonicotinoids reduces honey bee health near corn crops. *Science* **356**, 1395-1397 (2017).

9. Woodcock, B., Bullock, J. M., Shore, R. F., Heard, M. S., Pereira, M. G., Redhead, J., Ridding, L., Dean, H., Sleep, D., Henrys, P., Peyton, J., Hulmes, S., Hulmes, L., Sárospataki, M., Saure, C., Edwards, M., Genersch, E., Knäbe, S. & Pywell, R. F. Country-specific effects of neonicotinoid pesticides on honey bees and wild bees. *Science* **356**, 1393-1395 (2017).

10. Osterman, J., Wintermantel, D., Locke, B., Jonsson, O., Semberg, E., Onorati, P., Forsgren, E., Rosenkranz, P., Rahbek-Pedersen, T., Bommarco, R., Smith, H. G., Rundlöf, M. & de Miranda, J. R. Clothianidin seed-treatment has no detectable negative impact on honeybee colonies and their pathogens. *Nat. Comm.* **10**, 692 (2019).

11. Nazzi, F. & Conte, Y. L. Ecology of Varroa destructor, the Major Ectoparasite of the Western Honey Bee, Apis mellifera. *Annu. Rev. Entomol.* **61**, 417-432 (2016).

12. Grozinger, C. M. & Flenniken, M. L. Bee viruses: Ecology, pathogenicity, and impacts. *Annu. Rev. Entomol.* **64**, 205-226 (2019).

13. Johnson, R. M. Honey bee toxicology. *Annu. Rev. Entomol.* **60**, 415-434 (2015).

14. Bernd, H. The mechanisms and energetics of honeybee swarm temperature regulation. *J. Exp. Biol.* **91**, 25-55 (1981).

15. Brodschneider, R. & Crailsheim, K. Nutrition and health in the honeybee. *Apidologie* **41**, 278-294 (2010).

16. Mitchell, A. D., Mulhauser, B., Mulot, M., Mutabazi, A., Glauser, G. & Aebi, A. A worldwide survey of neonicotinoids in honey. *Science* **358**, 109-111 (2017).

17. Chauzat, M. P., Faucon, J.-P., Martel, A.-C., Lachaize, J., Cougoule, N. & Aubert, M.A Survey of Pesticide Residues in Pollen Loads Collected by Honey Bees in France. *J. Econ. Entomol.* **99**, 253-262 (2006).

18. Annoscia, D., Di Prisco, G., Becchimanzi, A., Caprio, E., Frizzera, D., Linguadoca, A., Nazzi, F. & Pennacchio, F. Neonicotinoid Clothianidin reduces honey bee immune response and contributes to Varroa mite proliferation. *Nat. Comm.* **11**, 5887 (2020).

19. Brutscher, L. M., Daughenbaugh, K. F. & Flenniken, M. L. Antiviral defense mechanisms in honey bees. *Curr. Opin. Insect Sci.* **10**, 71-82 (2015).

20. Berenbaum, M. & Johnson, R. Xenobiotic detoxification pathways in honey bees. *Curr. Opin. Insect Sci.* **10**, 51-58 (2015).

21. Bujok, B., Kleinhenz, M., Fuchs, S. & Tautz, J. Hot spots in the bee hive. *Naturwissenschaften* **89**, 299-301 (2002).

22. Nazzi, F., Brown, S. P., Annoscia, D., Del Piccolo, F., Di Prisco, G., Varricchio, P., Della Vedova, G., Cattonaro, F., Caprio, E. & Pennacchio, F. Synergistic Parasite-Pathogen Interactions Mediated by Host Immunity Can Drive the Collapse of Honeybee Colonies. *Plos Pathog.* **8**, e1002735 (2012).

23. Di Prisco, G., Cavaliere, V., Annoscia, D., Varricchio, P., Caprio, E.,Nazzi, F., Gargiulo, G. & Pennacchio, F. Neonicotinoid clothianidin adversely affects insect immunity and promotes replication of a viral pathogen in honey bees. *Proc. Natl. Acad. Sci. U.S.A.* **110**, 18466-18471 (2013).

24. Frizzera, D., Andreuzza, L., Boaro, G., D'Agaro, M., Del Fabbro, S., Zanni, V., Annoscia, D. & Nazzi, F. The interaction between a parasite and sub-optimal temperatures contributes to honey bee decline. *BioRxiv* doi: https://doi.org/10.1101/2021.07.22.453350 (2021).

25. Le Conte, Y., Arnold, G. & Desenfant, P. Influence of Brood Temperature and Hygrometry Variations on the Development of the Honey Bee Ectoparasite Varroa jacobsoni (Mesostigmata: Varroidae). *Environ. Entomol.* **19**, 1780-1785 (1990).

26. Breda, D. BeeStability, DOI:10.5281/zenodo.7050517

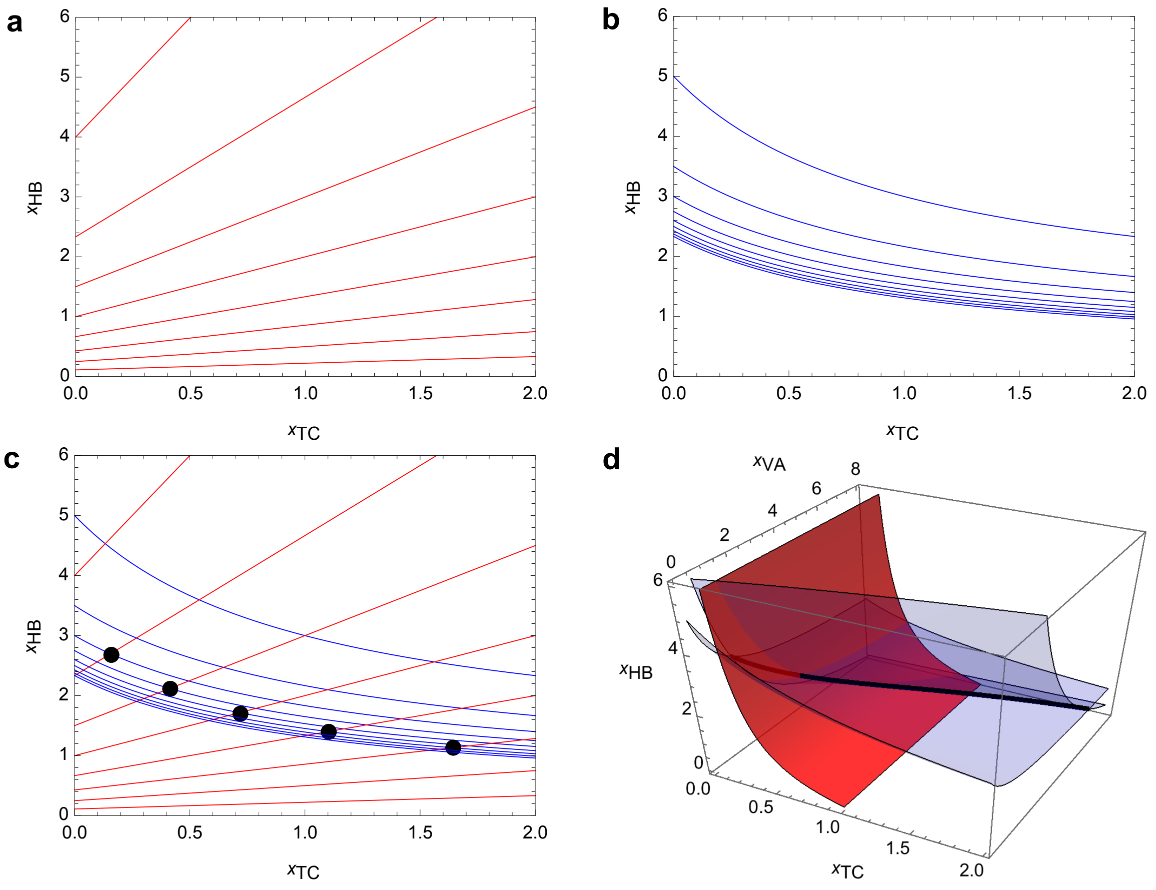

Supplementary Figure 3: **Uniqueness of equilibria for subsystem** (x_{HB}, x_{TC}, x_{VA})**.** (**a**) curves f_1 from the first equation of (12) for increasing x_{VA} top to bottom in the projected phase plane (x_{TC}, x_{HB}); (**b**) curves f_2 from (13) for increasing x_{VA} top to bottom in the projected phase plane (x_{TC}, x_{HB}); (**c**) intersection points (black dots) of f_1 (red) and f_2 (blue) for increasing x_{VA} top to bottom in the projected phase plane (x_{TC}, x_{HB}); (**d**) surfaces f_1 and f_2 (light blue), their intersection (thick black curve) and the surface from the second equation of (12) (red) in the phase space (x_{TC}, x_{VA}, x_{HB}): the unique equilibrium is the intersection point of the green curve with the red surface; see Supplementary Tab. **4** for the values of the parameters.
